# 30 YEARS OF THE MINERALOCORTICOID RECEPTOR: Mineralocorticoid receptor antagonists: 60 years of research and development

**DOI:** 10.1530/JOE-16-0600

**Published:** 2017-02-21

**Authors:** Peter Kolkhof, Lars Bärfacker

**Affiliations:** 1 Drug DiscoveryCardiology Research, Bayer AG, Wuppertal, Germany; 2 Drug DiscoveryMedicinal Chemistry, Bayer AG, Wuppertal, Germany

**Keywords:** mineralocorticoid receptor antagonists, spironolactone, canrenone, potassium canrenoate, eplerenone, apararenone, esaxerenone, finerenone

## Abstract

The cDNA of the mineralocorticoid receptor (MR) was cloned 30 years ago, in 1987. At that time, spirolactone, the first generation of synthetic steroid-based MR antagonists (MRAs), which was identified in preclinical *in vivo* models, had already been in clinical use for 30 years. Subsequent decades of research and development by Searle & Co., Ciba-Geigy, Roussel Uclaf and Schering AG toward identifying a second generation of much more specific steroidal MRAs were all based on the initial 17-spirolactone construct. The salient example is eplerenone, first described in 1987, coincidentally with the cloning of MR cDNA. Its launch on the market in 2003 paralleled intensive drug discovery programs for a new generation of non-steroidal MRAs. Now, 30 years after the cDNA cloning of MR and 60 years of clinical use of steroidal MRAs, novel non-steroidal MRAs such as apararenone, esaxerenone and finerenone are in late-stage clinical trials in patients with heart failure, chronic kidney disease (CKD), hypertension and liver disease. Finerenone has already been studied in over 2000 patients with heart failure plus chronic kidney disease and/or diabetes, and in patients with diabetic kidney disease, in five phase II clinical trials. Here, we reflect on the history of the various generations of MRAs and review characteristics of the most important steroidal and non-steroidal MRAs.

## Introduction

The history of mineralocorticoid receptor antagonists (MRAs) was initially a history of ‘aldosterone antagonists (AAs)’ as the identification of the first AAs during the 1950s was driven by the goal of identifying inhibitors of aldosterone activity in animals and humans. At that time, the main role of aldosterone was recognized as the control of renal sodium and potassium excretion, although the far-sighted Hans Selye called the mineralocorticoids ‘prophlogistic’ (Selye 1955). In 1942, he reported that desoxycorticosterone (as the acetate, DOCA), a precursor of aldosterone that was crystallized a decade later, induces nephrosclerosis accompanied by cardiac hypertrophy in animals (Selye 1942). Later, Selye found that application of one of the first AAs, spironolactone, protected rats from aldosterone-induced cardiac necrosis (Selye 1960). This was in the year when the spironolactone was launched as a potassium-sparing diuretic and the pioneering work of Selye on experimental aldosterone-induced inflammation, and fibrosis was not clinically considered for decades.

Years later, but still before the coding DNA of the mineralocorticoid receptor (MR) was cloned, it became clear that AAs block a specific receptor protein that has high affinity not only for aldosterone but also for cortisol in humans and corticosterone in rats and mice. Accordingly, AAs were called MRAs. Edelman and coworkers proposed a first model explaining the hormone-receptor activity of adrenal steroid hormones (Feldman *et al.* 1972) extended later by Corvol and coworkers (Corvol *et al*. 1981); finally, the publication by Evans’ group in 1987 elegantly identified the coding DNA sequence of the intracellular receptor protein that binds different mineralocorticoids and glucocorticoids (Arriza *et al.* 1987) with high affinity.

The 60 years of MRA research and development comprised three major waves within the pharmaceutical industry: The first basically took place within a single company, Searle Laboratories, which identified steroid-based spirolactone as the first anti-mineralocorticoids shortly after the purification of aldosterone. The second wave (still before the cloning of MR) was driven by the goal of identifying much more specific steroidal anti-mineralocorticoids, with the main active companies Searle, Ciba-Geigy, Roussel Uclaf and Schering AG. A decade after the cloning of MR, and ~50 years after Selye’s seminal work on the role of aldosterone in experimental renal and cardiac fibrosis, several pharmaceutical companies initiated drug discovery campaigns with the ultimate goal of identifying novel non-steroidal MRAs with defined pharmacokinetic and pharmacodynamic properties for use as safe and efficacious drugs for a broad spectrum of diseases.

One of the fascinating features of these 60 years is that the first MRAs were all discovered and characterized by *in vivo* experiments in animals and humans, whereas the discovery of novel non-steroidal MRAs employed high-throughput screening (HTS) of millions of compounds in several pharmaceutical companies. Such HTS campaigns for potent and selective MRAs were not possible before cloning of all cDNAs of the members of the steroid hormone-receptor family and their subsequent recombinant expression.

Here, we briefly summarize some characteristics of the most important steroidal and non-steroidal MRAs, including a historical perspective.

## Steroidal MRAs (the first 45 years of MRA R&D)

### Spironolactone

Information relating to the project rationale and synthesis efforts which culminated in the discovery of spirolactones (i.e. steroids that contain either a γ-lactone or a γ-hydroxy acid function at C-17) at Searle is rather sparse (Sturtevant 1992, Garthwaite & McMahon 2004). One chemistry program at Searle was originally focused on ‘cardioregulatory agents specifically for treating cardiac arrhythmias’ (Fitzgerald & Fitzgerald 2009), probably based on the attempt to combine parts of the steroidal structures of digitoxin and of progesterone (Garthwaite & McMahon 2004). The reason for mimicking progesterone was straightforward: Thorn and Engel found progesterone to be natriuretic in dogs (Thorn & Engel 1938) and Landau and coworkers confirmed this natriuretic activity of progesterone in men (Landau *et al.* 1955).

**Figure 1:**
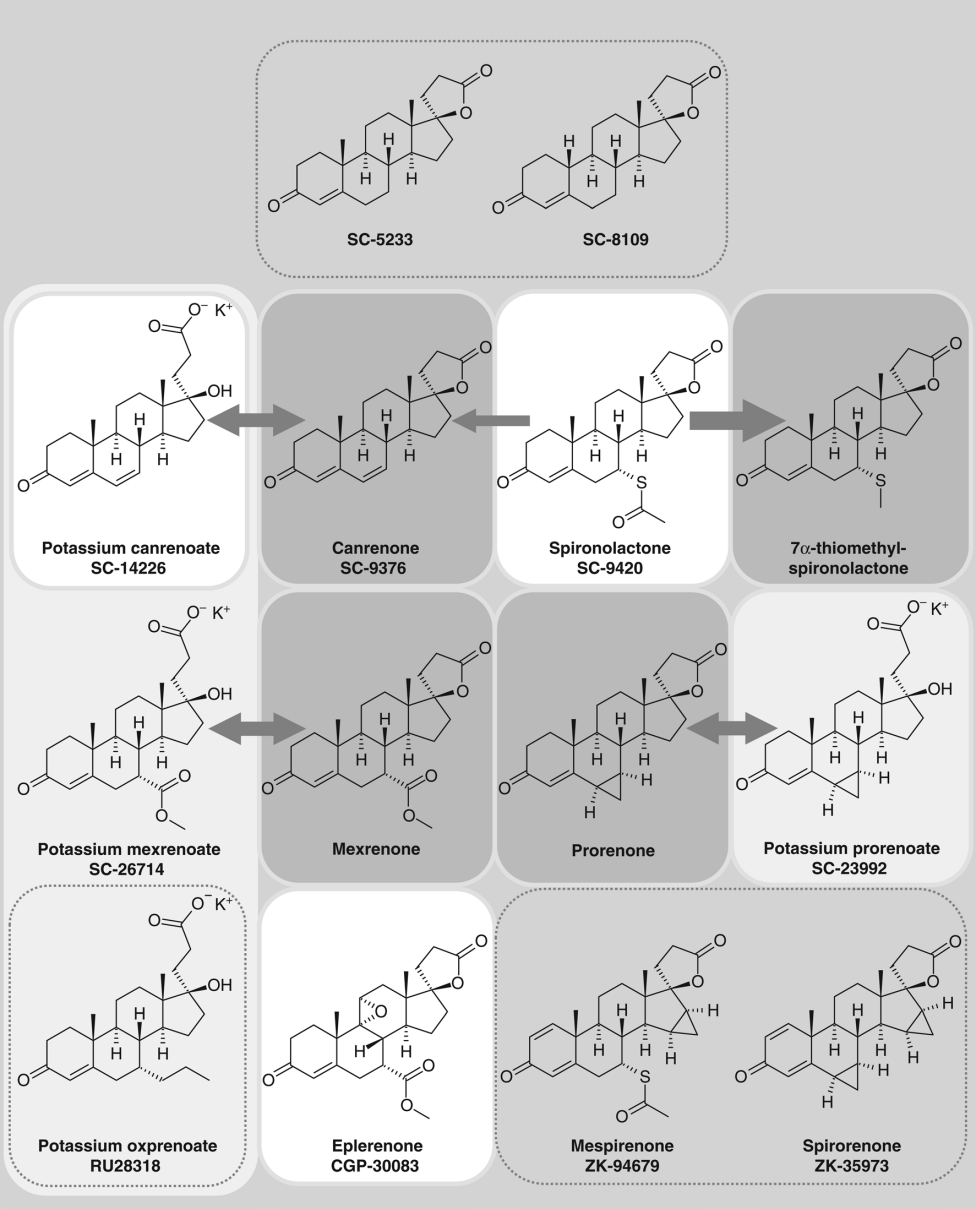
Important steroidal MRAs. Chemical structures of the most important 17-spirolactone derivatives, which were discovered and published between 1957 (beginning on top of the figure) and 1987 are shown. Launched drug compounds are highlighted by a white background. Open-ring potassium salt derivatives are highlighted by a light grey background (note that potassium canrenoate is both, a launched drug and a potassium salt derivative). Active metabolites are highlighted by a darker grey background. Arrows indicate either the generation of respective active metabolites from spironolactone (to different quantitative amounts, indicated by respective arrow sizes) or the equilibrium of the open-ring potassium salts with the respective lactone metabolite. Note the structural similarities of several stacked derivatives, e.g. mexrenone and eplerenone, or spironolactone and mespirenone.

After discontinuation of the original chemistry program by the Searle management (for unknown reasons) two biology groups within Searle independently investigated the spirolactone-based compound series in their own established biological assay systems, which were focused on the activity of mineralocorticoids (Sturtevant 1992). The group of Frank Sturtevant explored the compounds in a DOCA-dependent hypertension model, whereas Charles Kagawa, working in the renal labs of Gordon Van Arman, was examining the effects of the compounds in his rat assay of mineralocorticoid-dependent sodium retention (Cella & Kagawa 1957). A particular compound, SC-5233 (3-(3-oxo-17β-hydroxy-4-androsten-17α-yl)propionic acid γ-lactone; Fig. 1), demonstrated convincing antagonistic efficacy, which was strictly dependent on the presence of a mineralocorticoid in the models (Kagawa *et al.* 1959). Moreover, Kagawa and coworkers characterized the compound as a competitive antagonist of aldosterone according to the law of mass action (Kagawa *et al.* 1957). Sturtevant and Kagawa obviously convinced the management of Searle to initiate clinical trials with the compound (Sturtevant 1992, Fitzgerald & Fitzgerald 2009). Grant Liddle of Vanderbilt University Medical Center reported the natriuretic activity of SC-5233 in a patient with congestive heart failure and in a patient with Addison’s disease. SC-5233 was shown to be natriuretic in the patient with Addison’s disease (on a high-sodium diet) only in the presence of DOCA demonstrating that the compound is effective only in the presence of a sodium-retaining steroid, either endogenous or exogenous. Liddle’s clinical data and Kagawa’s preclinical data were submitted as manuscripts to the journal science in 1957 on August 19 and July 30 respectively and published head to head in one volume of Science in 1957 (Kagawa *et al.* 1957, Liddle 1957). In the preclinical paper, Kagawa and coworkers described the 19-nor analog of SC-5233, SC-8109 (Fig. 1), to be more potent than SC-5233 on the mineralocorticoid-dependent sodium retention in adrenalectomized rats. Hertz and Tullner subsequently reported that both compounds show substantial *in vivo* progestational activity in the Clauberg assay in estrogen-primed immature rabbits (Hertz & Tullner 1958) pointing to two different target tissues: the renal tubular apparatus and the endometrium. In the same year, Landau and Lugibihl (1958) published that progesterone is an endogenous antagonist of aldosterone and Liddle coincidentally published the clinical effects of five steroidal derivatives of SC-5233 in edematous patients (Liddle 1958).

The first steroidal spirolactones (Cella & Kagawa 1957) had only a sufficient exposure in animals and humans when administered parenterally. Therefore, John Cella and his chemistry team of Searle & Co. introduced chemical modifications with the goal to improve oral bioavailability. They found in a first step that dehydrogenation of saturated bonds leading to Δ^4,6^-3-oxo- and Δ^1,4,6^-3-oxo-derivatives all possessed enhanced oral activity (Cella & Tweit 1959). They then added, in a second step, a 7α-acetylthio group (among other modifications), as they already knew that the respective 7α-acethylthio-derivative of 17α-hydroxyprogesterone was more potent than the parent compound when administered parenterally (Cella & Tweit 1959). The corresponding 7α-acetylthio-17α-hydroxy-3-oxopregn-4-ene-21-carboxylic acid γ-lactone, SC-9420, exhibited oral bioavailability with a 46-fold higher potency than SC-5233 and was later introduced as spironolactone or Aldactone (Fig. 1) and administered to edematous patients who had not responded to the available standard therapy in a broad dose range up to 2400 mg/day (Garthwaite & McMahon 2004). A first conference on the clinical use of spirolactones and respective data in individual patients were held in Chicago on October 16, 1958 under the chairmanship of Irwin C. Winter and sponsored by G.D. Searle & Co. The compiled results of spironolactone in individual patients were sent to the FDA in September 1959, and spironolactone was launched in 1960 as a diuretic for the management of edematous conditions, primary aldosteronism and essential hypertension.

To date, the term ‘spironolactone’ has been mentioned in more than 8000 articles in PubMed, and several reviews have covered the basic pharmacology and pharmacokinetic properties of spironolactone (Sica 2005, Kolkhof & Borden 2012, Yang & Young 2016). The clinical use of spironolactone has also been extended to several indications independent of its ‘diuretic’ activity, especially after the re-discovery of the experimental pro-inflammatory and fibrotic activities of aldosterone originally described by Selye. The best known example is probably heart failure after Pitt and coworkers demonstrated mortality reduction with a rather low dose of spironolactone given on top of standard of care among patients with severe heart failure in the RALES landmark trial (Pitt *et al.* 1999); the actual MR ligand in these patients is cortisol, rather than aldosterone, reflecting the absence of the cortisol-converting enzyme 11β-hydroxysteroid dehydrogenase type 2 (11β-HSD2) in cardiomyocytes (Funder 2005). Other examples of spironolactone’s efficacy in preclinical models of stroke and small clinical trials of CKD let to euphoric editorials calling spironolactone a ‘renal aspirin’ (Bomback *et al.* 2009) or even ‘the Holy Grail’ (Dorrance 2008). On the other hand, there are two major issues associated with the use of spironolactone. The first, binding to the androgen receptor may cause painful gynecomastia and impotence, and binding to the progesterone receptor may cause menstrual irregularities. The second is the risk of developing potentially life-threatening hyperkalemia, especially among patients with reduced kidney function, when given in addition to other blockers of the renin-angiotensin system (RAS).

### Canrenone, potassium canrenoate and 7α-thiomethyl-spironolactone

Spironolactone can be considered not only as an MRA but also as a pharmacologically active prodrug: it has a short plasma half-life of less than 2 h and is metabolized in the liver to three major metabolites: Two sulfur-containing metabolites, 7α-thiomethyl-spironolactone (TMS, Fig. 1) and 6β-hydroxy-7α-thiomethyl-spironolactone (HTMS) and the major dethioacetylated metabolite canrenone (Fig. 1A), which have mean half-lives in healthy volunteers of 13.8, 15.0 and 16.5 h, respectively (Karim 1978, Garthwaite & McMahon 2004). Canrenone (Aldadiene, SC-9376), which was long considered to be the major active metabolite of spironolactone, and potassium canrenoate (Soldactone), the potassium salt of the γ-hydroxy acid derivative of canrenone (Fig. 1), are also marketed as own drug formulation in several countries, e.g. Belgium and Italy. However, TMS was subsequently found to be the main metabolite at steady state after oral application of 100 mg spironolactone per day: peak absolute and relative plasma concentrations of spironolactone, canrenone, TMS and HTMS were found to be 80 (10.3), 181 (23.3), 391 (50.3) and 125 (16.1) ng/mL (%) respectively (Gardiner *et al.* 1989). Nevertheless, it is remarkable how ‘spironolactone focused’ several textbooks of pharmacology are as the previously mentioned active metabolites are the major contributors of the pharmacological effects after administration of the prodrug spironolactone *in vivo*.

Gochman and Gantt first described the generation of canrenone after spironolactone application to a single healthy volunteer already in 1962 (Gochman & Gantt 1962). Canrenone is the common metabolite of both spironolactone and potassium canrenoate (SC-14266), the ring opened carboxylic acid form (Fig. 1). Potassium canrenoate is the only MRA, which is clinically available as solution for parenteral administration. Nevertheless, despite the structural difference between spironolactone and potassium canrenoate and perhaps because of the common generation of canrenone as an active metabolite, spironolactone and potassium canrenoate are often discussed in the literature as if they are identical compounds. We refer here to two important aspects of both compounds: pharmacokinetic including metabolism and toxicological profile.

The first pharmacokinetic investigations including the determination of metabolites after application of spironolactone in man were conducted by Karim & Brown (1972) and Sadée and coworkers (Sadée *et al*. 1973), and thoroughly summarized by Karim (1978) and Overdiek & Merkus (1987). However, quantitative determination of canrenone in these early studies was limited by a nonspecific fluorometric assay, subsequently improved by the establishment of an HPLC assay. Using this methodology, it was demonstrated that canrenone plasma concentrations were 5 times higher after potassium canrenoate administration than the same dose of spironolactone (Dahlöf *et al.* 1979). In contrast to the high proportion of canrenone generation after potassium canrenoate application, the canrenone proportion of the metabolites generated after spironolactone administration contributes only to about 15% after a single dose (Merkus *et al.* 1983) and about 23% after multiple doses (Gardiner *et al.* 1989). These results demonstrate that spironolactone and potassium canrenoate are very different in their metabolism, especially because a number of important sulfur-containing metabolites, i.e. TMS and HTMS, are not generated after potassium canrenoate administration. The elucidation of the different metabolism was also triggered by different toxicological findings with both compounds as outlined below.

The label of Aldactone tablets contains a black box warning, which indicates that spironolactone has been shown to be tumorigenic in chronic toxicity studies in rats (Aldactone NDA 2008). In fact, the origin of this particular safety warning has an interesting history, which includes an admonishing article in the New York Magazine in 1977 also (Vonder Haar & Miller 1977). There were substantial concerns about the safe use of spironolactone in chronic diseases and accordingly a subsequent decline in prescription rates for about a decade until the late 1980s. Briefly, the chronic safety profile of spironolactone was questioned with the first report of increased numbers of benign testicular adenomas in male rats that received a high dose (500 mg/kg) of spironolactone over a period of 78 weeks in 1974 (Mayer *et al.* 1988). In a second 104-week rat study using 10, 30 and 100 mg/kg/day of spironolactone, significant increases in hepatocellular adenomas and benign testicular and thyroid adenomas were observed (Aldactone NDA 2008).

Although no tumorigenic or carcinogenic potential was observed with spironolactone in chronic dog and monkey studies in that period (Lumb *et al.* 1978), additional studies with canrenoate in the early 1980s demonstrated a clear tumor association in rats: Sprague–Dawley rats received potassium canrenoate in doses of 30, 90 and 270 mg/kg/day over a period of 52 weeks in the so-called Hatano study in Japan during 1981. More than 50% of the animals in the 270 mg/kg group died due to myelogenous leukemia, tumors of the mammary gland and other neoplasms (Mayer *et al.* 1988). The study was repeated in the United States with rats receiving 20, 50, 125 and 270 mg/kg over a period of 104 weeks and myelogenous leukemia, tumors of the mammary gland and other carcinomas were statistically significantly increased at a dose of 125 mg/kg and higher (Mayer *et al.* 1988). Such tumors were not found with potassium canrenoate in toxicology studies performed in dogs and monkeys. Based on these findings with canrenoate, the Licensing Authority in the U.K. directed manufactures to stop recommending spironolactone for the treatment of hypertension and idiopathic edema in 1988. Accordingly, Spiroprop, a fixed-dose combination of spironolactone and propranolol for treating hypertension was withdrawn (Drug and Therapeutic Bulletin 1988). This led to a thorough reinvestigation of the metabolism of spironolactone and canrenoate, and Searle scientists resolved the phenomenon of the different toxicological findings: potassium canrenoate is metabolized to different epoxy-canrenone derivatives, which were found to be direct mutagens in the mouse lymphoma assay. Importantly, these mutagenic metabolites or their precursor epoxides were not formed from spironolactone (Cook *et al.* 1988, Oppermann *et al.* 1988). Therefore, the occurrence of myelocytic leukemia in long-term studies with canrenoate in rats can be explained by the formation of these mutagenic metabolites, whereas the generation of benign adenomas by very high doses of spironolactone is most probably related to endocrine pharmacological effects (Mayer *et al.* 1988).

### Potassium prorenoate/prorenone and potassium mexrenoate/mexrenone

Spironolactone use can be associated with characteristic adverse symptoms such as gynecomastia, which were first described in a letter to *The Lancet* in 1962 (Smith 1962). Spironolactone possesses clinical progestational and antiandrogenic activity due to interactions with the androgen and progesterone receptors (Huffman *et al.* 1978, Corvol *et al.* 1975, Jeunemaitre *et al.* 1987). Therefore, further drug discovery programs within Searle, Ciba-Geigy, Roussel Uclaf and Schering AG were initiated in the 1970s to identify much more specific steroidal MRAs. These programs were also influenced and dependent on novel techniques developed by academic groups, which enabled more direct *in vitro* investigation of modulating compounds 15 years prior to the cloning of the MR cDNA. Edelman’s group isolated and partially purified renal nuclear proteins with postulated mineralocorticoid receptor properties in 1968 (Herman *et al.* 1968). Using a rat kidney slice technique, Funder and coworkers could follow intracellular binding of aldosterone to a receptor protein in a time-dependent manner (Funder *et al.* 1972) and established in 1974 a structure–activity relationship (SAR) among 24 spirolactone derivatives by incubating such preparations of rat kidney slices with ^3^H-aldosterone and the different steroidal MRAs (Funder *et al.* 1974). They discovered that the affinity of spirolactones for the (not yet cloned) receptor is decreased by γ-lactone ring opening (with the formation of the water-soluble K^+^ salt), B ring unsaturation at the C6/C7 position and by γ-lactone unsaturation. In contrast, affinity was found to be markedly increased by esterification, or thio-esterification, at the C7 position in the B ring (Funder *et al.* 1974). This was a groundbreaking academic SAR work that supported the parallel medicinal chemistry investigations within the pharmaceutical companies.

It is remarkable that the departments of biological and chemical research of Searle came up with two novel steroidal MRAs almost 20 years after the first series of spironolactones: potassium prorenoate (SC-23992, 3-(17β-hydroxy-6β-7β-methylen-3-oxo-4-andosten-17α-yl)propionate), the potassium salt of the open lactone corresponding to prorenone (Fig. 1) and potassium mexrenoate (SC-26714, 3-(17β-hydroxy,7α-methoxycarbonyl-3-oxo-4-andosten-17α-yl)propionate) with the corresponding mexrenone (Fig. 1). Prorenoate was found to be 4.6 times more potent than spironolactone *in vivo* in the Kagawa rat assay using aldosterone as an agonist (Hofmann *et al*. 1975). As Funder and coworkers (Funder *et al*. 1976) found coincidentally that prorenoate has a lower affinity in androgen radioreceptor assays than spironolactone, prorenoate was considered as potential spironolactone successor with fewer antiandrogenic side effects (Funder *et al.* 1976). Claire and coworkers investigated the cellular mode of action and found that [^3^H]-labeled prorenone (the active metabolite of prorenoate) blocks nuclear translocation of the (yet-to-be cloned and fully purified) aldosterone receptor (Claire *et al.* 1979).

Results of a head-to-head comparison of prorenoate with spironolactone in fludrocortisone-challenged healthy volunteers were published in 1975 (Ramsay *et al.* 1975). As a single dose, prorenoate induced significantly more pronounced antikaliuretic activity than spironolactone; results of head-to-head comparison after repeated doses were published in 1982 (McInnes *et al.* 1982). This study showed that prorenoate was 3.6-fold more potent than spironolactone for urinary sodium excretion and 4.1-fold for elevating plasma potassium in otherwise healthy volunteers. This observation on plasma potassium was the focus of another multiple-dose study among healthy men taking the diuretic metolazone (McInnes *et al.* 1984). This study showed an estimated potency of prorenoate relative to spironolactone of 5.6-fold in attenuating metolazone-induced hypokalemia.

The second potential spironolactone successor from Searle was potassium mexrenoate (SC-26714). Dose-related natriuretic responses in animals indicated that potassium mexrenoate was between 2.1 (dog) and 4.5 (rat) times as potent as spironolactone (Hofmann *et al.* 1977). Although the corresponding lactone mexrenone played an important role later as a structural basis for eplerenone (see below) the development of potassium prorenoate and mexrenoate was not further undertaken by Searle.

### Potassium oxprenoate, spirorenone and mespirenone

Roussel Uclaf identified the open E-ring 7α-alkyl spirono­lactone potassium oxprenoate (potassium (7α,17α)-17-hydroxy-3-oxo-7-propylpregn-4-ene-21-carboxylic acid, Fig. 1). The groups of Corvol and Ménard in Paris investigated this MRA in humans (Ulmann *et al.* 1985). Oxprenoate was tested in comparison with spironolactone or placebo given as single-dose application in healthy volunteers challenged by the application of 9α-fluorohydrocortisone, aldosterone or of furosemide (to stimulate the endogenous RAAS). No significant difference was apparent between potassium oxprenoate and spironolactone in all three studies with respect to the urinary Na^+^/K^+^ ratio demonstrating that RU 28318 has an antimineralocorticoid effect identical to that produced by the same molar dose of spironolactone. Although not developed further for clinical purposes, the compound, which is actually much better known in the literature as RU28318, is still used in preclinical research due to its relatively good solubility in aqueous solutions enabling, for instance, intracerebroventricular administration in animal studies (Gomez-Sanchez *et al.* 1992).

Schering AG discovered 17α-spirolactones with a 15, 16-β-methylene modification. This series contained the most potent steroidal MRAs known to date, among them spirorenone (Bittler *et al.* 1982, Seifert *et al.* 1982, Casals-Stenzel *et al.* 1984) and mespirenone (Losert *et al.* 1986). Spirorenone (ZK 35973, (3-(17β-hydroxy-6β-7β-15β-16β-dimethylene-3-oxo-1,4-androstadiene-17α-yl, Fig. 1) was found to possess *in vivo* activity on the urinary Na^+^/K^+^ ratio on average 8.6 times higher than spironolactone in rats although its affinity for rat renal cytoplasmatic MR preparations was only 0.73 times the affinity of spironolactone (Casals-Stenzel *et al.* 1984). Spirorenone showed practically no affinity for the rat androgen receptor, but was found to have higher affinity for the progesterone receptor from estrogen-primed rabbit uterus than spironolactone (Laurent *et al.* 1983). Single-dose administration of spirorenone to healthy individuals demonstrated that spirorenone was at least 4 times as potent as spironolactone with respect to urinary sodium excretion (Seifert *et al.* 1982). It was found that spirorenone generates an active metabolite, 1,2-dihydro-spirorenone in humans (Krause *et al.* 1983); this compound was later named drospirenone and introduced to the market as the first potent synthetic progestogen exhibiting both antiandrogenic and antimineralocorticoid activity (Muhn *et al.* 1995). Like spirorenone, it was found to be eight times more potent than spironolactone on the MR in rats (Losert *et al.* 1985). The development of spirorenone as an MRA, on the other hand, was discontinued.

In search of compounds with increased antimineralocorticoid potency, reduced antiandrogenic potency and reduced or at least unchanged progestogenic potency compared with spironolactone, chemists at Schering AG identified mespirenone (ZK 94679, 15β,16β-methylene-spironolactone, Fig. 1, Losert *et al.* 1986). The compound was tested for its natriuretic efficacy vs spironolactone in healthy volunteers and found to be 6-fold more potent (Losert *et al.* 1986). Although it reached phase II clinical trials, it was discontinued in 1989.

### Eplerenone

Medicinal chemists at Ciba-Geigy synthesized 9-11α-epoxyderivatives of Searle’s spironolactone, canrenone, prorenone and mexrenone (de Gasparo *et al.* 1987) including 9-11α-epoxymexrenone, which was later renamed eplerenone (Fig. 1). This epoxy modification of the steroidal backbone forces the molecule in a more concave form thereby reducing the affinity for the other 3-oxo-steroid receptors AR and PR thus yielding more selective steroidal MRAs (Grob *et al.* 1997). However, eplerenone’s improved selectivity goes along with a relatively low *in vitro* affinity for MR, which is about 40-fold lower than spironolactone (Garthwaite & McMahon 2004, Hu *et al.* 2005, Fagart *et al.* 2010). *In vivo*, eplerenone compensates for this lower *in vitro* potency at MR with a higher fraction of bioavailable compound, whereas spironolactone is bound 94% to human plasma proteins (Chien *et al.* 1976) and eplerenone’s bound fraction is only about 50% (Inspra Prescribing Information 2002). Eplerenone was investigated in clinical phase I studies in a head-to-head manner with spironolactone published in 1989 (de Gasparo *et al.* 1989). It demonstrated natriuretic activity after fludrocortisone challenge in heathy volunteers with a rough equi-efficacy at 50 mg to 25 mg spironolactone. The large-scale production of eplerenone was obviously difficult and costly and the compound passed from Ciba-Geigy to Searle, to Monsanto, to Pharmacia and ultimately to Pfizer (Ménard 2004).

Weinberger and coworkers (Weinberger *et al*. 2002) showed that 100 mg eplerenone (the maximal approved daily dose for the treatment of hypertension in the United States) given once-daily (OD) or 50 mg twice-daily (BID) to hypertensive patients had an efficacy of 50–75% compared to 50 mg spironolactone twice daily. These results are in line with the only other published head-to-head trial with spironolactone and eplerenone among patients with hypertension and evidence of primary aldosteronism (Parthasarathy *et al.* 2011). Eplerenone was subsequently investigated in a pivotal clinical phase III in subjects with heart failure after several phase II trials in patients with hypertension. EPHESUS (Eplerenone Post-Acute Myocardial Infarction Heart Failure Efficacy and Survival Study) randomized 6642 patients within 14 days of acute myocardial infarction to either eplerenone (mean dose-equivalent was 42.6 mg) or placebo on top of standard of care including ACEIs/ARBs, beta-blockers, diuretics and aspirin. After a mean follow-up of 16 months, the primary endpoint, death from any cause or hospitalization for CV events, was significantly reduced by eplerenone (Pitt *et al.* 2003). This reduction was solely attributed to the prevention of sudden cardiac death. The incidence of serious hyperkalemia (≥6 mmol/L) was significantly higher in the eplerenone group vs the placebo group (*P* = 0.002). Accordingly, eplerenone was launched in the United States for the treatment of chronic heart failure after myocardial infarction in 2002, more than 40 years after spironolactone (Garthwaite & McMahon 2004, Ménard 2004).

The pharmacokinetic–pharmacodynamic relationship of eplerenone is remarkable and might serve as a role model for MRAs (Kolkhof & Borden 2012). Eplerenone has no active metabolites and a much shorter half-life than spironolactone’s active metabolites: Cook and coworkers (Cook *et al*. 2003) determined a half-life of 4 h in steady state after multiple dose applications of 100 mg (which is the approved dose of eplerenone in the U.S. for hypertension). Weinberger and coworkers (Weinberger *et al*. 2002) demonstrated that the blood pressure-lowering efficacy of eplerenone in hypertensive patients is higher when given twice daily than once daily. Thosar and coworkers (Thosar *et al*. 2003) demonstrated a natriuretic effect for ~10 h after a single oral dose of 50 mg eplerenone (the approved OD dosage in HF), and a concomitant short half-life of only 2.9 h at this dose in healthy subjects. However, despite possessing such a short half-life, eplerenone provides mortality reduction when administered once daily to HF patients (Pitt *et al.* 2003, Zannad *et al.* 2011). Therefore, it might be possible that some pharmacodynamic effects mediated by MRAs (e.g. blood pressure control or serum potassium changes) are the consequence of a significant drug exposure over a long period (long half-life, AUC driven), whereas others (e.g., anti-inflammatory, -hypertrophic and -fibrotic effects) are the consequences of relatively short drug exposure (short half-life, *C*_max_ driven) triggered by different signaling cascades.

## Non-steroidal MRAs (the recent 15 years of MRA R&D)

There was a remarkable rise in basic research and small clinical trials on MRAs in several pathophysiological conditions beyond the traditional role of aldosterone in renal sodium/potassium homeostasis since the late 1980s. Weber and coworkers found a marked effect of aldosterone on collagen synthesis in intra-myocardial coronary arteries in the rat (Brilla *et al.* 1990); Pearce and Funder demonstrated the expression of MR in the heart (Pearce & Funder 1987), and in the vasculature (Funder *et al*. 1989). Rocha and coworkers (Rocha *et al*. 2002) identified vascular inflammation as a potential mechanism of aldosterone-mediated myocardial injury in rats receiving aldosterone chronically in addition to a sodium load, more than 40 years after Selye’s seminal work on the role of mineralocorticoids in renal and cardiac fibrosis (Selye 1946). These results stimulated further preclinical and clinical studies on inflammatory and fibrotic diseases including chronic kidney disease, among others. Another trigger for additional R&D activities on novel MRAs was the issue of hyperkalemia, which was an obligate effect of MR blockade by steroidal MRAs, particularly when given with other RAS blockers to ‘real-life’ patients, typically with variable degrees of concomitant kidney dysfunction (Juurlink *et al.* 2004, Svensson *et al.* 2004, Dinsdale *et al.* 2005). Interestingly, Taylor and Faloon in 1959 predicted that *‘…the possibility of initiating potassium intoxication with this regimen (i.e. spironolactone plus potassium supplementation in cirrhotic patients) should be borne in mind’* (Taylor & Faloon 1959), and individual cases of marked hyperkalemia associated with the use of spironolactone were published early on (Herman & Rado 1966, Pongpaew *et al.* 1973, Feinfeld & Carvounis 1978). Accordingly, several pharmaceutical companies initiated drug discovery campaigns with the ultimate goal of identifying novel non-steroidal MRAs with novel physicochemical properties potentially reducing the hyperkalemia risk. We focus here briefly on three novel non-steroidal MRAs, apararenone, esaxerenone and finerenone, which are in several late stage clinical trials.

### Apararenone

**Figure 2:**
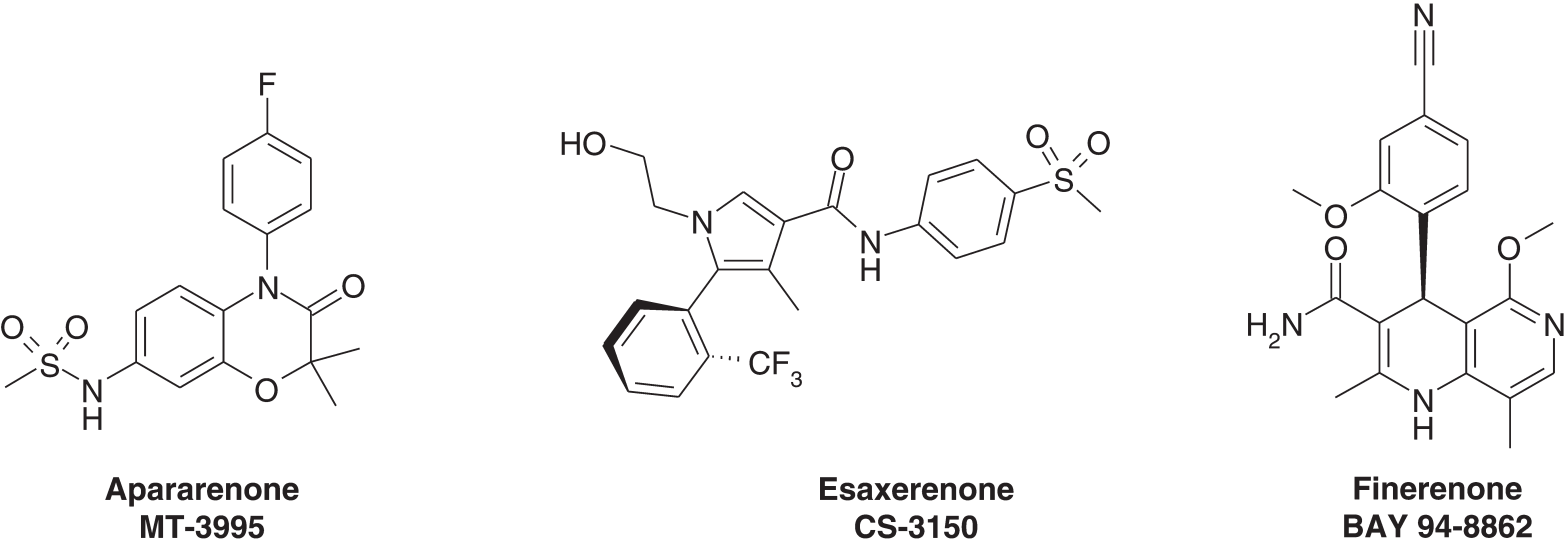
Novel non-steroidal MRAs in clinical development. Chemical structures of the three clinically most advanced non-steroidal MRAs apararenone, esaxerenone and finerenone are shown (from left to right).

Unfortunately, no published preclinical or clinical data are currently available for the non-steroidal MRA apararenone (MT-3995, N-[4-(4-fluorophenyl)-2,2-dimethyl-3-oxo-3,4-dihydro-2H-1,4-benzoxazin-7-yl]methanesulfonamide, Fig. 2). We can only refer here to publicly available information from the data bank (clinicaltrials.gov): Mitsubishi Tanabe Pharma was conducting apararenone for the treatment of diabetic nephropathy (DN) in phase II studies in Eastern Europe and Japan and is currently recruiting for an extended treatment study (28 weeks) in patients with DN (excluding patients with an urinary albumin to creatinine ratio (UACR) of ≥300 mg/g). Recently, a phase II clinical trial has been initiated among patients with non-alcoholic steatohepatitis (NASH) in Japan (Table 1).

**Table 1:** Current late stage clinical studies with non-steroidal MRAs.

NCT#	Study completion	Phase	No. enrolled	Locations	Study name/condition
**Apararenone**					
NCT2923154	Oct 2017	Phase 2	40	Japan	An exploratory study of MT-3995 in patients with non-alcoholic steatohepatitis (NASH)
NCT2676401	Apr 2018	Phase 2	280	Japan	An extended treatment study of MT-3995 in patients with diabetic nephropathy
**Esaxerenone**					
NCT2885662	Dec 2017	Phase 3	40	Japan	A study of CS-3150 to evaluate efficacy and safety in patients with primary aldosteronism
NCT2808026	Dec 2017	Phase 3	20	Japan	A study to evaluate efficacy and safety of CS-3150 in Japanese patients with severe hypertension
NCT2807974	Dec 2017	Phase 3	50	Japan	A study of CS-3150 to evaluate efficacy and safety in hypertensive patients with type 2 diabetes and albuminuria
NCT2807987	Dec 2017	Phase 3	50	Japan	A study of CS-3150 to evaluate efficacy and safety in combination with ARB or ACE inhibitor in hypertensive patients with moderate renal impairment
NCT2848170	Dec 2017	Phase 3	40	Japan	Phase 3 study to examine the relation between antihypertensive effect and baseline factors exploratively, compared to olmesartan medoxomil in patients with essential hypertension
NCT2722265	Dec 2017	Phase 3	360	Japan	Open-label, multicenter, interventional, dose titration study to assess the long-term study of CS-3150 2.5 mg and 5 mg alone as monotherapy or in combination with other antihypertensive drug in Japanese patients with essential hypertension
NCT2890173	Dec 2017	Phase 3	930	Japan	A double blind study of CS-3150 to evaluate efficacy and safety compared to eplerenone in patients with essential hypertension (ESAX-HTN Study)
**Finerenone**					
NCT2545049	Feb 2019	Phase 3	6400	Global	A randomized, double-blind, placebo-controlled, parallel-group, multicenter, event-driven phase 3 study to investigate efficacy and safety of finerenone on the reduction of cardiovascular morbidity and mortality in subjects with type 2 diabetes mellitus and the clinical diagnosis of diabetic kidney disease in addition to standard of care (FIGARO-DKD)
NCT2540993	May 2019	Phase 3	4800	Global	A randomized, double-blind, placebo-controlled, parallel-group, multicenter, event-driven phase 3 study to investigate the safety and efficacy of finerenone, in addition to standard of care, on the progression of kidney disease in subjects with type 2 diabetes mellitus and the clinical diagnosis of diabetic kidney disease (FIDELIO-DKD)

### Esaxerenone

Esaxerenone (CS-3150, (5S)-1-(2-hydroxyethyl)-4-methyl-N-[4-(methylsulfonyl)phenyl]-5-[2-(trifluoromethyl)phenyl]-1H-pyrrole-3-carboxamide, Fig. 2) is a non-steroidal MRA, which was discovered by Exelixis and out-licensed to Daiichi Sankyo in 2006. The receptor profile of the compound was published by Arai and coworkers (Arai *et al*. 2015*a*): Esaxerenone more potently blocked aldosterone-induced transcriptional activation of human MR in a cell-based assay with an IC_50_ value of 3.7 nM compared to spironolactone and eplerenone, with IC_50_ values of 66 and 970 nM respectively. Esaxerenone also showed at least a 1000-fold higher selectivity for MR over other steroid hormone receptors *in vitro*, a long half-life and a more pronounced antihypertensive activity induced by DOCA/salt-loading to rats than spironolactone or eplerenone (Arai *et al.* 2015*a*). Chronic treatment of Dahl salt-sensitive hypertensive rats demonstrated a dose-dependent antihypertensive effect of esaxerenone with an equivalent reduction of blood pressure with a low dose of esaxerenone (0.5 mg/kg) to that of spironolactone (100 mg/kg) and eplerenone (100 mg/kg). Similarly, esaxerenone also reduced proteinuria and renal hypertrophy with superior potency to that of spironolactone and eplerenone, which demonstrated only partial cardiorenal protection in this study (Arai *et al*. 2015*b*). Furthermore, esaxerenone not only prevented but also ameliorated hypertension and renal injury in DOCA rats (Arai *et al.* 2016).

In January 2015, Daiichi Sankyo announced the start of two different phase II studies: a dose-finding study in Japanese patients with type 2 diabetes mellitus (T2DM) and microalbuminuria and a study to evaluate the efficacy and safety of esaxerenone in Japanese patients with hypertension. Although no data from these studies have been published so far in a scientific journal, some preliminary clinical data were presented recently in an investor presentation published on Daiichi Sankyo’s web site (Daiichi Sankyo’s FY2016 Q2 earnings call, November 1, 2016). In September 2016, Daiichi Sankyo announced it is undertaking a phase 3 pivotal trial (ESAX-HTN) to evaluate esaxerenone as a treatment for essential hypertension in Japanese patients. The primary objective is non-inferiority of an antihypertensive effect in comparison to eplerenone (50 mg) in a 12-week study among ~930 patients. Several smaller local studies with blood pressure determinations as primary outcome parameter in different patient populations are currently recruiting patients (Table 1).

### Finerenone

Several reviews have already summarized preclinical and clinical data of finerenone (Kolkhof *et al.* 2015, 2016, Liu *et al.* 2015, Bramlage *et al.* 2016, Gomez-Sanchez 2016, Haller *et al.* 2016, Yang & Young 2016), and we will highlight here only some specific features of this novel non-steroidal MRA.

A cluster of dihydropyridines (DHPs) acting as MRAs *in vitro* were identified in an ultrahigh throughput screening program of ~1,000,000 compounds at Bayer (Ergueden *et al.* 2005). This finding was very surprising as DHPs constitute the known class of L-type calcium channel blockers (CCB) like nifedipine. Chemistry programs within Bayer, Pfizer and Merck led to potent and specific MRAs with and without additional L-type calcium channel activity (Brandish *et al.* 2009, Arhancet *et al.* 2010, Fagart *et al.* 2010, Bärfacker *et al.* 2012).

Chemical optimization of DHP-based compounds led to a novel series of heterobicyclic analogs of naphthyridine derivatives (Bärfacker *et al.* 2012). The dihydronaphthyridine finerenone (previous nomenclature BAY 94-8862, Fig. 2) was identified as a potent MRA with excellent selectivity vs all other steroid hormone receptors as well as 65 important receptors and ion channels including the L-type Ca^2+^ channel (Bärfacker *et al.* 2012, Pitt *et al.* 2012). Thus, finerenone is at least as potent as spironolactone and even more selective (at least 500-fold toward MR) than eplerenone. Table 2 shows that finerenone more potently blocks MR in a functional cell-based assay system than spironolactone and eplerenone independent of MR agonist (i.e. aldosterone, cortisol, coticosterone or DOCA). Finerenone was shown not only to block wild-type MR *in vitro* but also the gain-of-function S810L MR mutant (Amazit *et al.* 2015), which is the cause for early-onset hypertension in men and gestational hypertension in women (Geller *et al.* 2000). Progesterone and both steroidal MRAs, spironolactone and eplerenone, paradoxically activate this S810L MR mutant (Geller *et al.* 2000, Amazit *et al.* 2015). Using an automated HTS microscopy of MR subcellular distribution, it was demonstrated that finerenone delays aldosterone-induced nuclear translocation MR more efficiently than spironolactone (Amazit *et al.* 2015). Moreover, chromatin immunoprecipitation (ChIP) assays showed that finerenone and spironolactone differentially affect the recruitment of the transcriptional cofactor SRC-1 on a MR target promoter. Although finerenone acts as an inverse agonist, i.e., reducing SRC-1 recruitment even in the absence of aldosterone at the promoter of the SCNN1A gene, spironolactone acts as a partial agonist, i.e., promoting SRC-1 recruitment but to a lesser extent as aldosterone (Amazit *et al.* 2015). These results show that structurally different MRAs can lead to a different pharmacology. In contrast to spironolactone and eplerenone, the non-steroidal MRAs BR-4628 and finerenone are ‘bulky-passive’ antagonists (Fagart *et al.* 2010, Bärfacker *et al.* 2012, Amazit *et al.* 2015). Binding of ‘bulky’ non-steroidal MRAs probably cause a protrusion of helix 12 in MR’s C-terminal-activating function 2 domain, and as a consequence, an unstable receptor–ligand complex, which is unable to recruit coregulators (Kolkhof & Borden 2012).

**Table 2:** *In vitro* potency of spironolactone, eplerenone and finerenone vs different MR agonists in a functional cell-based MR assay.

Agonist	**Antagonist**		
	Spironolactone IC_50_ (nM)	Eplerenone IC_50_ (nM)	Finerenone IC_50_ (nM)
Aldosterone	24	990	18
Cortisol	19	360	5
Corticosterone	41	940	24
DOCA	114	1970	46

Half-maximally inhibitory concentrations (IC_50_) of MRAs were determined in a functional cell-based transactivation assay based on CHO-K1 cells stably expressing the ligand binding domain of MR as described by Fargart and coworkers (Fargart *et al*. 2010). The IC_50_ values determined vs aldosterone as agonist were taken from Pitt and coworkers (Pitt *et al*. 2012). All other IC_50_ values of the three MRAs were determined in parallel investigations performed in duplicate. The agonist concentrations used were 1 nM for aldosterone and corticosterone and 10 nM for cortisol and DOCA. The GraphPad Prism software (version 3.02, GraphPad Software) was used for curve fitting and calculation of IC_50_ values.

The non-steroidal structure of finerenone does not only influence the binding mode within MR, but especially determines the physicochemical properties like lipophilicity and polarity, which have a strong impact on plasma protein binding, transport, tissue penetration and distribution. The steroidal MRAs are 6- to 10-fold more lipophilic than finerenone, whereas the latter exhibits higher polarity than the steroidal MRAs (Kolkhof *et al.* 2015). Quantitative whole-body autoradiography with [^14^C]-labeled finerenone demonstrated a balanced distribution of finerenone into cardiac and kidney tissues of healthy rats, which is in clear contrast to the respective distribution pattern of spironolactone and eplerenone in rodents (Kolkhof *et al.* 2014). Experiments using radioactively labeled eplerenone demonstrated at least a 3-fold higher accumulation of the drug equivalent concentration in the kidneys compared with heart tissue in rats, whereas high drug equivalent concentrations (original drug plus metabolites) after administration of radioactively labeled spironolactone were detected within the kidneys, but the whole radioactivity in cardiac tissue was below the detection limit in a similar study in mice (reviewed in Kolkhof & Borden 2012).

Finerenone reduced cardiac hypertrophy, pro-B-type natriuretic peptide (BNP) and proteinuria more efficiently than eplerenone when directly comparing equinatriuretic doses (Kolkhof *et al.* 2014). Finerenone reduced systolic blood pressure (SBP) values in DOCA/salt rats only at the highest investigated dose of 10 mg/kg, although almost all functional parameters were improved with the dose of 1 mg/kg that had no significant influence on blood pressure. Systolic blood pressure in animals treated with 10 mg/kg finerenone was significantly lower in comparison to animals treated with 100 mg/kg eplerenone in a head-to-head manner. In a mouse model of pressure overload-induced HF treatment with finerenone compared head-to-head with eplerenone resulted in a more pronounced prevention of myocardial hypertrophy (Grune *et al.* 2016). These head-to-head observations contributed to the hypothesis that use of finerenone is either associated with a more pronounced anti-hypertrophic/anti-fibrotic efficacy at a given comparable risk for developing hyperkalemia or with a reduced risk of developing hyperkalemia at a given comparable anti-hypertrophic/-fibrotic efficacy (Bauersachs 2013, Naegele *et al.* 2016). To test this hypothesis, finerenone was tested head-to-head vs spironolactone among patients with chronic heart failure and concomitant CKD (Pitt *et al.* 2012). It was found that once-daily oral administration of 5 and 10 mg of finerenone were at least as effective as spironolactone (25 or 50 mg/day) in decreasing BNP, NT-pro-BNP and urinary albumin, but were associated with significantly lower increases in serum potassium, a significantly lower incidence of hyperkalemia as well as a lower incidence of worsening renal function (Pitt *et al.* 2013). However, the reduction in SBP and the rise in serum aldosterone levels in patients receiving finerenone 5 and 10 mg OD were smaller than those observed in patients receiving spironolactone in ARTS.

Available data from five clinical phase II trials with finerenone in more than 2000 patients with HF and additional CKD and/or diabetes as well as in patients with diabetic kidney disease have demonstrated so far that neither hyperkalemia nor reductions in kidney function were limiting factors to its use. Recently, finerenone demonstrated a nominally improved outcome including death and CV hospitalization compared to eplerenone in a phase IIb trial with 1066 heart failure patients with reduced ejection fraction (HFrEF) and concomitant T2DM and/or CKD (Filippatos *et al.* 2016). Different dose strengths of finerenone were also investigated in 823 randomized patients with T2DM and diabetic kidney disease receiving standard of care (SoC) (i.e., ACEIs/ARBs) and either once-daily (OD) finerenone or placebo (Bakris *et al.* 2015). Addition of finerenone to SoC resulted in dose-dependent, significant reductions in albuminuria at doses of 7.5, 10, 15 and 20 mg after 90 days of treatment (Bakris *et al.* 2015). As nocturnal hypertension is common in patients with CKD, a *post hoc* study of finerenone for its effect on nocturnal hypertension in a subset of patients of the ARTS-DN study in whom ambulatory blood pressure measurement (ABPM) data were available were conducted. Patients with nocturnal hypertension showed a more pronounced reduction in nocturnal BP with finerenone (absolute change of −9.5 mmHg in 10 mg and −12.0 mmHg in the 20 mg dose group respectively) than under placebo (−2.9 mmHg) at day 90 (Ruilope *et al*. 2016). However, only an appropriate trial among patients with essential hypertension should give definitive answers on the blood pressure-lowering potency and efficacy of finerenone in hypertensive patients.

Overall, the ARTS-HF and ARTS-DN studies demonstrated the best benefit-to-risk ratio at doses of 10–20 mg finerenone OD. Accordingly, finerenone at daily doses of 10 and 20 mg is currently being investigated in two large outcome trials in patients with DKD (FIGARO-DKD, Nbib2545049 and FIDELIO-DKD and Nbib2540993) (Table 1).

## Conclusions

**Figure 3:**
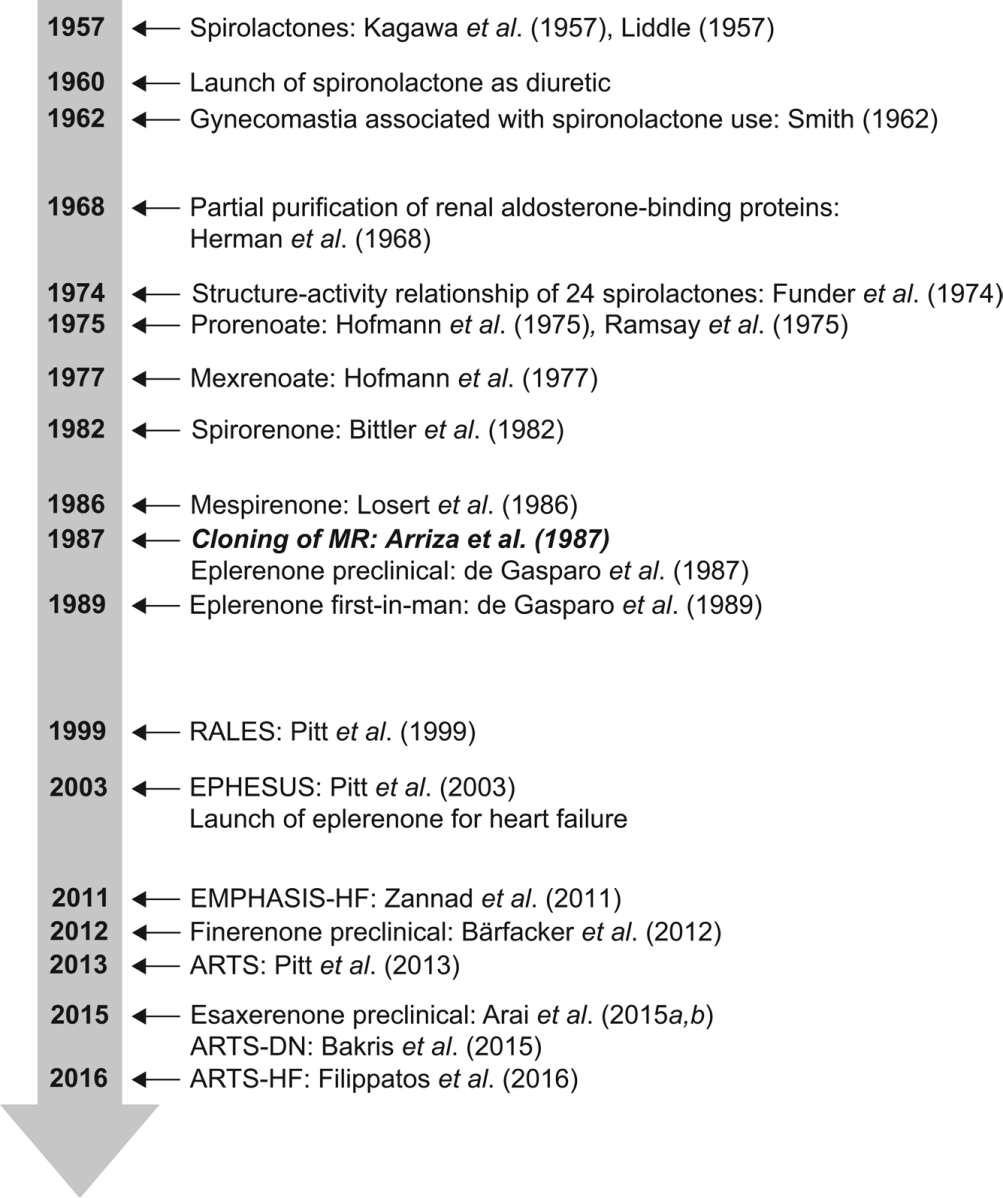
60 years of research and development on MRAs. The time bar highlights relevant publications on the discovery of MRAs or important clinical trial results with MRAs (RALES, EPHESUS, EMPHASIS-HF, ARTS, ARTS-DN and ARTS-HF) in a given year. Note that cloning of MR was at midway within the 60 years of R&D on MRAs.

Joel Ménard once highlighted the remarkable timeframe of almost 45 years between the first clinical usage of spironolactone and the market launch of its steroidal successor eplerenone (Ménard 2004). This is even more remarkable when considering the structural similarities of these two compounds. A real paradigm change was initiated with the search for non-steroidal MRAs as these will ultimately lead to compounds with different physicochemical, pharmacokinetic and eventually pharmacological properties in comparison to the 17-spirolactone-based MRAs including spironolactone and eplerenone. The long way of R&D activities from the initial spirolactones to the current non-steroidal MRAs is briefly summarized in Fig. 3. The de Gasparo paper from 1989 summarizing the successful discovery story including the first-in-man study of eplerenone ended with two remarkable sentences: *‘The clinical development of antialdosterones is lengthy and costly, and it is possible that the advantages of this treatment ... appears for many clinicians to be more theoretical than practical. If this is true, spironolactone would remain a unique compound, not only because it was the first anti-hormone used in clinical medicine, but also because it would have no successor’* (de Gasparo *et al.* 1989). Now, almost 30 years later, it is clear that the development of any new cardiovascular drug has become a billion dollar investment, but the advantages of MRA use for a broad spectrum of patients are far beyond theoretical.

## Footnote

This paper is part of a thematic review section on 30 Years of the Mineralocorticoid Receptor. The guest editors for this section were John Funder and Maria Christina Zennaro.

## Declaration of interest

Dr Peter Kolkhof and Dr Lars Bärfacker are full time employees of Bayer AG.

## Funding

This work did not receive any specific grant from any funding agency in the public, commercial, or not-for-profit sector.
